# PhyLIS: A Simple GNU/Linux Distribution for Phylogenetics and Phyloinformatics

**DOI:** 10.4137/ebo.s3169

**Published:** 2009-07-30

**Authors:** Robert C. Thomson

**Affiliations:** Center for Population Biology and Department of Evolution and Ecology, University of California, Davis, CA 95616 USA. Email:rcthomson@ucdavis.edu

**Keywords:** linux, phylogenetics, phyloinformatics, operating system

## Abstract

PhyLIS is a free GNU/Linux distribution that is designed to provide a simple, standardized platform for phylogenetic and phyloinformatic analysis. The operating system incorporates most commonly used phylogenetic software, which has been pre-compiled and pre-configured, allowing for straightforward application of phylogenetic methods and development of phyloinformatic pipelines in a stable Linux environment. The software is distributed as a live CD and can be installed directly or run from the CD without making changes to the computer. PhyLIS is available for free at http://www.eve.ucdavis.edu/rcthomson/phylis/.

## Introduction

Phylogenetic methods are playing a growing role in nearly all fields of biology. As researchers accumulate data, the size of phylogenetic analyses and the scope of inferences for which they are employed have both increased dramatically.^1^ Increasingly, researchers employ a diverse array of methods that have been developed by a large and talented group of biologists and programmers. While the availability of these methods is an obvious boon for biology as a whole, the sheer number of software packages that are now regularly employed in phylogenetic research forces biologists and system administrators to spend growing amounts of time installing, configuring and maintaining software rather than focusing on research.

The availability of cheap multi-core processors exacerbates this issue, and now many even-moderately sized labs routinely build small clusters or groups of several phylogenetic workstations. Phyloinformatic research, in particular, depends largely on processing power and highly parallel analyses that are spread across many computers and processors. As phyloinformatic pipelines become more complex and sophisticated, careful standardization of operating systems across computers also becomes more complex. While this growing computational power gives researchers the ability to be more creative in attempting complex and time-consuming analyses, the process of compiling, installing, and configuring software for these machines becomes increasingly repetitive, error-prone, and time-consuming. This problem is, in principle, easily solved. What is needed is a simple, stable platform that is specifically geared toward performing phylogenetic analyses. Perhaps the simplest (from a user perspective) solution to this need is a lightweight Linux-based operating system, geared specifically toward phylogenetic and phyloinformatic research. Existing Linux distributions such as Bio-Linux^2^ and SciBuntu^3^ represent useful steps in this direction. However, these distributions are aimed at a more general usership, and thus do not incorporate many of the packages that are now standard tools for phylogenetic analysis. Moreover, because phylogenetic methods are currently experiencing rapid development, there is a need for a distribution specifically focusing on this area. PhyLIS aims to fill this need.

## Implementation

PhyLIS v1.0 is a free GNU/Linux distribution based on the popular and user-friendly Ubuntu Linux distribution. The name PhyLIS is an acronym for Phylogenetic Linux for Informatics and Systematics. The distribution comes with most commonly used phylogenetic software pre-compiled, installed, and configured, which allows this software to be executed by simply typing the appropriate command (Table 1). PhyLIS also contains popular scripting languages (and appropriate phyloinformatic packages) including Perl (with BioPerl), Python (with BioPython), and R (with several packages). It implements parallel versions of several particularly processor-intensive programs using MPI (including BEST,^4^ MrBayes,^5^ and raxML).^6^ PhyLIS aims to present a complete phylogenetic workbench for all steps of analysis from sequence data manipulation to alignment and tree search, including visualization (for alignments and trees), model selection, divergence time estimation, macroevolutionary analyses and tools for automation and batch analysis.

The distribution intentionally re-uses most of the system maintenance packages from Ubuntu, making the actual use of the operating system very similar to Ubuntu (and Debian, upon which Ubuntu is based). Overall, the non-phylogenetic aspects of PhyLIS (e.g. installation, updating software, file system structure) have been kept as close as possible to Ubuntu in order to preserve the large amount of development that the Ubuntu team has put into ensuring an easy-to-use operating system. Because of this, navigating, updating and using the operating system is largely intuitive for users that are already familiar with more widely used operating systems. The bundled phylogenetic tools (Table 1) are available via the command line interface, allowing for straightforward batch analyses and scripting. Software that employs a graphical user interface can be run using graphical launchers on the desktop, in addition to the command line.

## Installation

PhyLIS is distributed as a live CD and can be used in two ways. First, it can be booted from the CD without making changes to the underlying operating system. This is useful, for example, for temporarily employing computers (which may not be configured for phylogenetics, or may be configured for other purposes) to run phylogenetic analyses when not in use for their primary purpose. At the completion of the analysis, the results can be transferred to permanent storage and the machine rebooted, restoring it to its previous configuration and operating system. The live CD mode is also useful for testing PhyLIS with little time commitment before deciding whether to install it.

Second, PhyLIS can be directly installed from the live CD using a simple graphical installer that allows for a new, complete installation (erasing the previous operating system), or a partitioned installation (allowing for dual boot systems). The distribution has been tested and can be installed on most 32- and 64-bit PCs (including Apple computers that use Intel processors).

## Conclusions

PhyLIS aims to simplify the process of carrying out complex phylogenetic analyses and has utility both for individual researchers and for teaching environments. The operating system presents a large suite of tools in a stable platform and should be useful for system administrators performing many installations. However, it is also simple enough to use that individual researchers with little previous Linux experience can employ it effectively. PhyLIS is under active development and undergoes periodic updates every six months to incorporate new versions of software and minor bug fixes. Users are encouraged to request additional software or features that would enhance the utility of the operating system; these will be incorporated into future releases of PhyLIS. The latest release is freely available at http://www.eve.ucdavis.edu/rcthomson/phylis.

## Figures and Tables

**Table 1:** Bundled phylogenetic software packages and commands used to call them.

Software package	Command
Ade4^7^	(R package, see documentation)
Ape^8^	(R package, see documentation)
Aptreeshape^9^	(R package, see documentation)
BEAST^10^	beast
BEAUTi^10^	beauti
BEST (including a parallel version)^4^	mbbest, mbbest_mpi
Bioperl^11^	(Perl package, see documentation)
Biopython^12^	(Python package, see documentation)
Blast2 package^13^	blast2, blastclust, blastall, megablast, etc.
Bucky^14^	bucky
Clustalw^15^	clustalw
Dialign2^16^	dialign2–2
FigTree^17^	figtree
Garli^18^	garli
Geiger^19^	(R package, see documentation)
Hmmer^20^	(See documentation)
jModelTest^21^	jmodeltest
LogAnalyser^17^	loganalyser
LogCombiner^17^	logcombiner
MAFFT^22^	mafft
Mesquite (graphical and headless versions)^23^	run_mesquite.sh, run_headless_mesquite.sh
MrBayes (including a parallel version)^5^	mb, mb_MpI
Muscle^24^	muscle
Ouch^25^	(R package, see documentation)
Paloverde^26^	paloverde
PhYLIp^27^	phylip
Phylocom^28^	phylocom
PhyML^29^	phyml
Physim^30^	(R package, see documentation)
Phyutility^31^	phyutility
PoA^32^	poa
R	R
r8s^33^	r8s
RaxML (including a parallel version)^6^	raxmlHPC, raxmlHPC_MpI
Readseq^34^	readseq
Seaview^35^	seaview
Seq-gen^36^	seq-gen
T-coffee^37^	t_coffee
Tracer^17^	tracer
TreeAnnotator^17^	treeannotator
TreeLogAnalyser^17^	treeloganalyser
